# The effect of a novel probiotic on metabolic biomarkers in adults with prediabetes and recently diagnosed type 2 diabetes mellitus: study protocol for a randomized controlled trial

**DOI:** 10.1186/s13063-016-1762-x

**Published:** 2017-01-09

**Authors:** Talia Palacios, Luis Vitetta, Samantha Coulson, Claire D. Madigan, Gareth S. Denyer, Ian D. Caterson

**Affiliations:** 1 The Boden Institute of Obesity, Nutrition, Exercise and Eating Disorders, University of Sydney, Sydney, Australia; 2 University of Sydney, Sydney Medical School, Sydney, Australia; 3 Medlab Clinical, Sydney, Australia; 4 School of Life and Environmental Sciences, University of Sydney, Sydney, Australia

**Keywords:** Gut microbiota, Prediabetes, Probiotics, Type 2 diabetes mellitus

## Abstract

**Background:**

Shifts in the gastrointestinal microbiome have been shown to contribute to the progression of metabolic diseases including prediabetes and type 2 diabetes mellitus. Research suggests that *in-vivo* modulation of the gut microbiome by specific probiotic microorganisms may improve insulin sensitivity and blood sugar management, preventing or delaying the development of type 2 diabetes mellitus. However, further research is needed to understand the effect of probiotics as a therapy for the treatment of metabolic diseases. An evidence-based multi-species probiotic was developed to encourage a shift in the gastrointestinal bacterial cohort from a disease-prone to a balanced state with the aim of improving metabolic markers associated with type 2 diabetes mellitus.

**Methods:**

Sixty adults with a body mass index ≥25 kg/m^2^ with prediabetes or type 2 diabetes mellitus (diagnosed within the previous 12 months) will be enrolled in a double-blind, placebo-controlled pilot study. Participants will be randomized to a multi-species probiotic or placebo for 12 weeks. Both groups will receive lifestyle and nutritional advice. The primary outcome measure is the change between groups in fasting plasma glucose levels from baseline to 12 weeks. Secondary outcome measures include, but are not limited to, the change in lipid profile, systemic inflammation, gut permeability, and faecal microbial and metabolomic profiles. Blood and stool samples are collected at baseline and 12 weeks after treatment.

**Discussion:**

Intentional manipulation of gastrointestinal microbial profiles may be useful for preventing and controlling type 2 diabetes mellitus and its associated metabolic complications.

**Trial registration:**

Australian New Zealand Clinical Trials Registry, ACTRN12613001378718. Registered on 16 December 2013.

**Electronic supplementary material:**

The online version of this article (doi:10.1186/s13063-016-1762-x) contains supplementary material, which is available to authorized users.

## Background

The relationship between the gastrointestinal microbiota and obesity-associated disorders has gained extensive research interest in the past 10 years. A disturbed gut microbiota expressed as gut dysbiosis (an intestinal physical barrier abnormality) has been associated with the progression and maintenance of obesity, type 2 diabetes mellitus, cardiovascular diseases and metabolic syndrome [1–5]. The mechanisms by which gut dysbiosis produces and influences these metabolic alterations are via regulation of the host’s energy balance and storage and by promoting endotoxaemia or bacteraemia [6]. Furthermore, postprandial blood glucose levels are very much influenced by the gut bacteria, with recent research demonstrating a profound influence on how individuals responded to identical food items that could be accurately predicted based on their gut bacterial profiles [7]. Gut dysbiosis may be restored to a balanced state through microbiota-based interventions, which may improve metabolic markers associated with type 2 diabetes mellitus through immunomodulatory and anti-inflammatory pathways.

A probiotic is a microbiota-based intervention defined as ‘a live microorganism, which, when administered in adequate amounts, confers beneficial health effects on the host’ [2, 8, 9]. A meta-analysis evaluating the effect of probiotics on glycaemia suggests that probiotics can play an important role in the prevention and treatment of type 2 diabetes mellitus [10]. Certain probiotic species have improved insulin sensitivity, inflammatory markers and lipid profiles in obese, type 2 diabetes mellitus and dyslipidaemic subjects [11–13]. However, it is not known whether a combination of probiotic species that have demonstrated beneficial therapeutic effects individually can improve metabolic markers associated with type 2 diabetes mellitus and have an additive effect to standard care. Therefore, we have formulated a novel prescription containing eight probiotic species that belong to the *Lactobacillus*, *Bifidobacterium*, *Streptococcus* and *Saccharomyces* genera to improve glucose metabolism in subjects with prediabetes and early type 2 diabetes mellitus*.* This multi-species probiotic formula has been tested previously in our laboratory using *in-vitro* models with rodent fat and muscle cell lines. The results from these *in-vitro* experiments showed the supernatants collected from the growth media of the probiotic decreased lipid accumulation in 3T3-L1 adipocytes and restored glucose uptake in insulin resistant L6 muscle cells [7]. The formulation and dosage proposed in this study have not been investigated previously in human studies. Therefore, the aim is to test the safety and efficacy of this novel probiotic formulation in adults with prediabetes and early type 2 diabetes mellitus. We hypothesize that a shift in the gut microbiome induced by this multi-species probiotic will decrease metabolic and inflammatory markers and result in improved blood glucose management.

## Methods

### Design

**Fig. 1:**
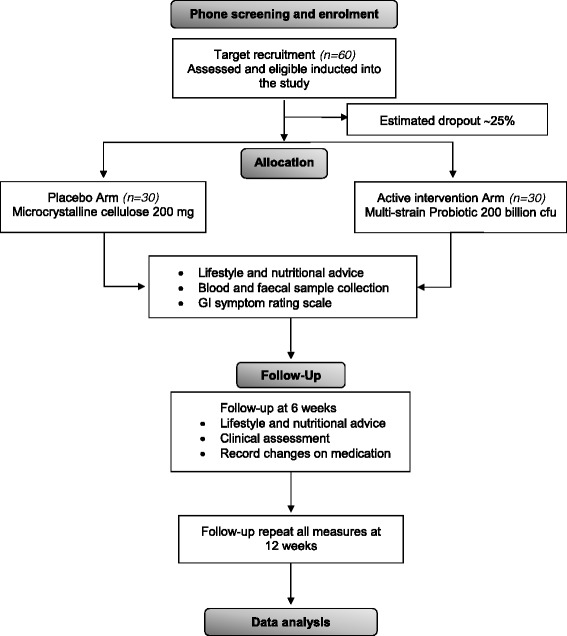
CONSORT flowchart of participants’ progress through the study

This pilot study is a single site, randomized, double-blind, placebo-controlled clinical trial conducted at the Charles Perkins Centre Royal Prince Alfred Clinic in Sydney, Australia. Sixty adults with prediabetes or early type 2 diabetes mellitus will be randomized to take either a multi-species probiotic capsule or placebo for 12 weeks. The Standard Protocol Items: Recommendations for Interventional Trials (SPIRIT) was used to elaborate the study protocol (see Additional file 1). Participants’ progression through the trial is presented in Fig. 1 (CONSORT diagram [14]).

### Recruitment process

Subject recruitment will be through the Boden Institute’s clinical trials register, the Sydney Local Health District intranet, the University of Sydney website and Diabetes Australia social media channels.

### Allocation

Participants will be randomized to the probiotic or placebo group without stratification using computer-generated random numbers (FileMarker Pro). Both participants and study investigators will be blinded to treatment allocation. Participant unblinding will only be requested in a medical emergency, where knowledge of the study treatment is essential for any treatment of the participant. The reason for unblinding will be documented and the study treatment will not be revealed to any member of the study team.

### Data handling and record keeping

Data is completed on case report forms, source documents (both written on paper and time and date stamped electronic capture) and entered into a database. This database will be password protected and backed up the University of Sydney server. All results will be sent to participants by email.

### Inclusion criteria

Participants are eligible for the study if they meet the following criteria:

- Aged ≥ 18 years
- Body mass index ≥ 25 kg/m^2^
- Have prediabetes or have been diagnosed with type 2 diabetes mellitus within the previous 12 months
- Are treated by diet alone or diet plus metformin
- Are willing to adhere to the study protocol (no yoghurt, fermented food, dietary supplements, probiotics or prebiotics) for the duration of the study.

The definition of prediabetes is based on the American Diabetes Association guidelines [15] and participants must have: (i) a fasting plasma glucose level between 5.6 mmol/l and 6.9 mmol/l or; (ii) two-hour post-challenge (oral glucose tolerance test) plasma glucose level between 7.8 mmol/l and 11 mmol/l or; (iii) HbA1c between 5.7% and 6.4%.

The criteria for the diagnosis of type 2 diabetes mellitus is based on the American Diabetes Association guidelines [15]. Participants are included in the study if they have been diagnosed with type 2 diabetes mellitus by their general practitioner in the previous 12 months or have: (i) a fasting plasma glucose level ≥ 7.0 mmol/l or; (ii) 2-hour post-challenge (oral glucose tolerance test) plasma glucose level ≥ 11.1 mmol/l or; (iii) an HbA1c ≥ 6.5%.

### Exclusion criteria

- Type 1 diabetes mellitus
- Type 2 diabetes mellitus diagnosed for longer than 12 months
- Taking anti-obesity drugs or blood glucose-lowering medications (i.e. sulfonylureas, alpha-glucosidase inhibitors, thiazolidinediones and glucagon-like peptide-1 analogues) other than metformin
- Concomitant gastrointestinal disorders (i.e., irritable bowel syndrome, inflammatory bowel disease and coeliac disease)
- Recent use (within the previous 4 weeks) of antibiotics and dietary supplements (fish oil, probiotics, prebiotics, multivitamins, minerals, nutraceuticals and herbal preparations)
- Pregnancy, breastfeeding or planning to become becoming pregnant
- Alcohol abuse or the use of any illicit drugs
- Clinical evidence of active infection or any severe illness unrelated to diabetes.

### Intervention and compliance

#### Investigational product

The study coordinator will obtain written informed consent and randomize eligible participants to the multi-species probiotic or placebo. The study product contains either 50 × 10^9^ colony forming units (cfu) of a multi-species probiotic (27 × 10^9^ cfu of *Lactobacillus* spp., 22.5 × 10^9^ cfu of *Bifidobacterium* spp., 450 × 10^6^ cfu of *Streptococcus* spp. and 45 × 10^6^ cfu of *Saccharomyces* spp., patent pending) or 50 mg of the placebo (maltodextrin) per capsule. The probiotic and placebo capsules are opaque white and look and smell identical. Verbal and written instructions on how to take the study product or placebo will be provided at the initial and each review visit. Enrolled participants will be required to:

- Take two capsules twice per day (20 min before breakfast and dinner) with cold non-carbonated water. The product is not to be mixed or taken with hot drinks or foods, as heat and stomach acids can reduce the stability of the probiotic bacteria
- Store the study product in the fridge at 4–6 °C
- Record the number of capsules taken each day in the study diary
- Bring all capsules remaining in the bottle to the next visit
- Avoid eating or drinking yoghurt, fermented food, dietary supplements (i.e. vitamins, minerals, nutraceuticals, herbal preparations, probiotics, prebiotics or fish oils) and antibiotics (unless recommended by a health professional).

#### Study product compliance

Capsule counting, at weeks 6 and 12, will be used to assess the participant’s compliance in taking the study product. A record of the date, the visit and the amount of study product dispensed and returned will be documented for each participant. Participants are defined as non-compliant if they have taken less than 80% of the study product on both occasions.

#### Lifestyle guidance

Subjects in both groups of the study will receive dietary and lifestyle advice from a research dietitian. This advice is according to the 2013 National Health and Medical Research Council dietary guidelines for Australian adults. The guidelines focus on improving fruit and vegetable intake, portion size control, reduction of energy-dense and low-nutrient density foods and drinks, encouraging physical activity by walking 10,000 steps per day and reducing sedentary behaviour. At baseline, participants will be provided with a pedometer to track the number of steps they take.

### Outcome measures

#### Primary outcome measure

The difference in any change in fasting plasma glucose level between the intervention and placebo groups between baseline and 12 weeks.

#### Secondary outcome measures

- The safety of the probiotic formula
- The change between groups in the following biomarkers or measurements from baseline to 12 weeks:
  (i) HbA1c
  (ii) insulin sensitivity indices, including Matsuda-ISI [16] and homeostatic model assessment-IR [17]
  (iii) fasting plasma lipids, such as triglycerides, free fatty acids, total cholesterol, HDL-c and LDL-c
  (iv) plasma zonulin and lipopolysaccharide
  (v) hs-CRP
  (vi) gastrointestinal symptoms
  (vii) body mass index, waist circumference, and waist and hip ratio
  (viii) blood pressure
  (ix) faecal microbial and metabolomics profiles.

### Participant timeline

**Fig. 2:**
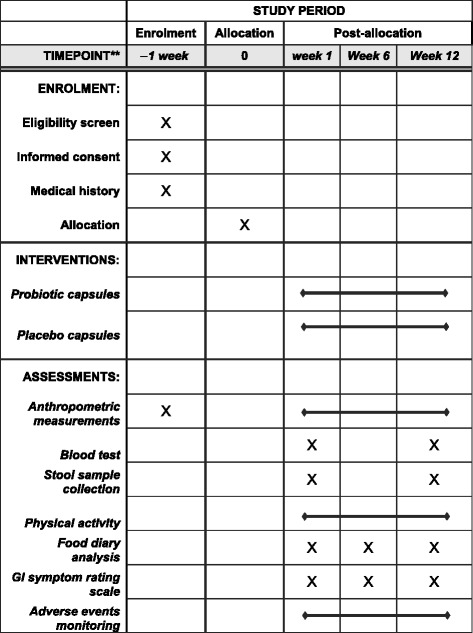
SPIRIT schedule of enrolment, interventions and assessments

Screening, intervention and assessment visits will be performed by a research nutritionist. The schedule of visits and measurements is given in Fig. 2 in compliance with SPIRIT guidelines.

### Data collection

#### Clinical examination

Medical history, prescribed and non-prescribed medications, alcohol intake and smoking habits will be recorded during the screening visit. Anthropometric measurements will be collected using standardized examination procedures [18] and calibrated equipment at three time points (baseline, 6 weeks and 12 weeks). Height will be measured using a stadiometer with an adjustable headpiece. Participants will be weighed wearing an examination gown and underwear only. Waist circumference will be measured at the midpoint between the lowest margin of the last rib and the top of the iliac crest. Hip circumference is measured around the widest portion of the buttocks.

Participants will record a 7 day food diary (5 working days and 2 weekend days) at baseline, then at 6 and 12 weeks after randomization. Food Works 8 will be used to assess energy and macronutrients including dietary fibre intake. Participants will wear a pedometer for the study duration. The Stanford Leisure-Time Activity Categorical Item (L-Cat) 2.2 physical activity questionnaire [19] will be completed at baseline, and 6 weeks and 12 weeks after treatment. The number of steps taken will be recorded daily in a study diary.

#### Blood samples

Blood samples will be collected after fasting overnight at baseline and 12 weeks. Participants who do not have type 2 diabetes mellitus will undergo a 75 g oral glucose tolerance test to assess glucose and insulin levels at three time points (0 min, 60 min and 120 min). Blood samples to measure glucose, insulin, HbA1c, lipids and hs-CRP will be sent to a commercial laboratory for analysis. Samples of 12 ml of blood collected in ethylenediaminetetraacetic acid (EDTA) plasma tubes will be used for lipopolysaccharide and zonulin analysis. These samples will be immediately placed at 4 °C and centrifuged for 15 minutes at 1000 *g* (or 3000 rpm) at 2–8 °C within 30 minutes. Plasma samples will be aliquoted in pyrogen-free tubes using pyrogen-free pipette tips and stored at −80 °C. Serum endotoxin will be measured using the Pierce™ Limulus Amoebocyte Lysate chromogenic endotoxin quantification kit (Thermo Fisher, Sydney, NSW, Australia). The EDTA-plasma zonulin concentration will be measured with the human haptoglobin ELISA kit (Abcam®, Sydney, NSW, Australia). Lipopolysaccharide and zonulin analyses will be performed in duplicate.

#### Stool samples

Stool samples will be collected at baseline and 12 weeks using a stool specimen collection kit. This collection kit includes an instruction booklet for the stool sample collection and transportation, ice packs, gloves, a sterile container, sealed plastic pouch, cool box and an AnaeroGen™ Compact sachet, which preserves the microbiological characteristics of the sample for 72 hours. Participants will bring the stool sample to the clinic and the containers will be stored at 4 °C and analyzed by matrix-assisted laser desorption ionization time-of-flight mass spectrometry 24–48 hours after collection. In addition, a 1 g sample will be stored at −80 °C for faecal microbial and metabolite profiling. DNA extraction will be performed using the QIAamp DNA stool kit (Qiagen, Sydney, NSW, Australia). Faecal microbial composition will be identified by sequencing the 16S rRNA using the Ion S5 next-generation sequencing system (Thermo Fisher, Sydney, NSW, Australia). Short-chain fatty acids, secondary bile acids and choline by-products will be measured using gas chromatography-mass spectrometry.

### Data analysis and statistical considerations

#### Sample size

As this is a pilot study, no evidence about blood glucose differences is available for this product. Therefore, it was decided to use the number of participants needed to detect a mean difference between the groups in fasting plasma glucose level of 2.0 mmol/l (standard deviation 2.0 mmol/l) at week 12 with 80% power and 95% confidence as a starting point. The number of subjects needed is 32. The fact that the study includes two different cohorts (participants with prediabetes and early type 2 diabetes mellitus) was also taken into consideration, as owing to the lack of stratification at randomization the variance could be high. Therefore, the sample size has been inflated to 30 per group (including a drop-out rate of 25%) to give a total sample size of 60 eligible participants.

#### Statistical analysis

Analysis will be conducted using the intention-to-treat principle and missing data will be imputed with baseline values for a conservative estimate (i.e. no change). Descriptive statistics will be presented as mean ± standard deviation, or median with range, as appropriate. Primary and secondary outcomes will be analyzed using generalized linear models. The following covariates will be added to the model: sex, age and percentage weight change. Additionally, if there is an imbalance between the groups of prediabetes and type 2 diabetes mellitus, this will be included as a covariate in the model. Three planned subgroup analyses will be completed between participants who are taking metformin, participants classified as prediabetic or type 2 diabetes mellitus and participants defined as compliant. Chi–square analysis will determine associations between categorical variables.

For the gut microbiome analysis, sequenced data will be interpreted using the bioinformatics tools programmed in the Ion Reporter™ software. Quantitative Insights Into Microbial Ecology (QIIME) algorithms will determine the bacterial diversity within a sample (alpha diversity) and between all the samples (beta diversity). Additionally, multivariate data analysis with principal component analysis on the diversity indexes and comparisons of genus and species level data will be performed to reveal differences in the microbial composition between the two groups. Differences in relative abundance of taxa between the intervention and placebo group and between participants receiving and not receiving metformin will be determined using ANOVA, using subject as a blocking factor. Changes in relative abundance will be tested for correlations with patient biochemical measurements. For all statistical tests, the Benjamini–Hochberg false discovery rate adjustment [20] will be used to account for the number of taxa tested in each comparison.

### Adverse events

Adverse events are defined as any unfavourable and unintended sign (including an abnormal laboratory finding), symptom, or disease (new or exacerbated) temporally associated with the use of the study product, whether or not considered related to the treatment. The study investigator will monitor each participant for adverse events during the study. All adverse signs or symptoms reported between consent and final follow-up will be recorded. Adverse events are reported descriptively by group.

#### Serious adverse events

All serious adverse events, related or not related to the study product, are recorded on paper and electronic case report forms. Serious adverse events will be reported in compliance with the requirements of the Sydney Local Health District Human Research Ethics Committee. Probiotic intake has not been associated with any major side effects and extensive safety data are available on their effects; however, participants will be discontinued from the study product if it is decided that a serious adverse event may be related to probiotic consumption.

### Ethical considerations

Ethical approval has been granted by the Sydney Local Health District Human Research Ethics Committee (Royal Prince Alfred Hospital). This study will be carried out according to the Declaration of Helsinki, the National Health and Medical Research Council National Statement on Ethical Conduct in Research Involving Humans and the Notes for Guidance on Good Clinical Practice as adopted by the Australian Therapeutic Goods Administration (2000) (CPMP/ICH/135/95) and the International Conference on Harmonisation Good Clinical Practise guidelines.

## Discussion

This randomized trial will assess the efficacy and safety of a multi-species probiotic formulation in the management of prediabetes and type 2 diabetes mellitus metabolic markers. The study outcomes may lead to novel treatments to reduce the metabolic disturbances associated with these disorders. This study will also provide empirical evidence to address currently unresolved issues with the efficacy and safety of probiotics. In designing this clinical study, several key decisions were made to overcome current limitations in the published literature and reduce possible biases.

While focusing on those with prediabetes, subjects with early type 2 diabetes mellitus were included to assess the feasibility of inducing partial or complete remission of type 2 diabetes mellitus. This decision also meant including participants taking metformin as this drug is commonly prescribed early in type 2 diabetes mellitus; it has also been shown to have effects on the microbiome [21, 22]. Furthermore, microbiota-based interventions may reduce gastrointestinal symptoms associated with metformin administration with a consequent improvement in medication compliance [23]. As far as we are aware, the interaction between the gastrointestinal microbiota, probiotics and metformin has not been explored in patients with prediabetes and type 2 diabetes mellitus. Therefore, this study will assess the efficacy and safety of a novel multi-species probiotic and provide preliminary data on its effect on metformin.

Limitations of the study include the short-term impact of the intervention, as subjects will be treated for only 12 weeks. Additionally, the small number of visits during the study can be considered a risk factor for non-compliance. Moreover, it must be remembered this study is investigating the short-term impact of a multi-species probiotic formulation to help improve fasting blood glucose levels. It is known that probiotics do not recolonize the intestinal tract but rather are transient colonizers and that wash-out of administered doses can take from approximately 4–6 weeks, thereby leading to a therapy that may be not be permanent.

This trial will provide pilot data of a novel probiotic formula that may shift the gastrointestinal microbial profile from a disease-prone to a balanced state and improve glucose metabolism. The findings will enhance the understanding of the role that probiotics may play on metabolic biomarkers in individuals with high glucose levels. Additionally, microbiome biomarkers associated with the risk of developing type 2 diabetes mellitus may be found by exploring the gut microbial and metabolomic profiles.

## Trial status

Participant recruitment started in August 2015 and is ongoing.

## Additional files

Additional file 1: SPIRIT 2013 checklist. (DOCX 51 kb)
Additional file 2: Participant information sheet and consent form for the study ‘Effect of a novel probiotic on metabolic biomarkers in adults with prediabetes and recently diagnosed with type 2 diabetes’. (DOCX 71 kb)

## Abbreviations

ANOVA: analysis of variance
cfu: colony forming unit
EDTA: ethylenediaminetetraacetic acid
ELISA: enzyme-linked immunosorbent assay
HbA1c: glycosylated haemoglobin
hs-CRP: high-sensitive C-reactive protein
L-CAT: Stanford Leisure-Time Activity Categorical Item
QIIME: Quantitative Insights Into Microbial Ecology
SPIRIT: Standard Protocol Items: Recommendations for Interventional Trials
