# Supplementary material for: The effect of a novel probiotic on metabolic biomarkers in adults with prediabetes and recently diagnosed type 2 diabetes mellitus: study protocol for a randomized controlled trial
Source: Trials. 2017 Jan 9;18:7. doi: 10.1186/s13063-016-1762-x (PMC5223589; doi:10.1186/s13063-016-1762-x)
Supplement: Additional file 2: — Participant information sheet and consent form for the study ‘Effect of a novel probiotic on metabolic biomarkers in adults with prediabetes and recently diagnosed with type 2 diabetes’. (DOCX 71 kb) [file 13063_2016_1762_MOESM2_ESM.docx]

Additional file 2: Participant information sheet and consent form for the Effect of a novel Probiotic on Metabolic Biomarkers in Adults with Pre-diabetes and Recently Diagnosed with Type 2 Diabetes


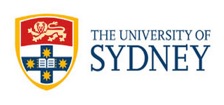


**Participant Information Sheet**

**Interventional Study**

The Boden Institute of Obesity, Nutrition, Exercise & Eating Disorders

| **Title** | The Effect of a Multistrain Probiotic on Metabolic Biomarkers in Adults with Pre-diabetes and Recently Diagnosed with Type 2 Diabetes |
| --- | --- |
| **Protocol Number** | X14-0369 |
| **Coordinating Principal Investigators** | Prof Ian Caterson  Prof Luis Vitetta |
| **Associate Investigator(s)** | Ms Talia Palacios  Dr Samantha Coulson  Dr Henry Butt |
| **Location** | Sydney |

1. **Introduction**

You are invited to take part in this research project because you have been recently diagnosed with pre-diabetes or type 2 diabetes. The research project is testing whether a supplement containing multiple probiotic strains will help to improve blood glucose.

This Participant Information Sheet/Consent Form tells you about the research project. It explains the tests and treatments involved. Knowing what is involved will help you decide if you want to take part in the research. Please read this information carefully. Ask questions about anything that you don’t understand or want to know more about. Before deciding whether or not to take part, you might want to talk about it with a relative, friend or your local doctor.

Participation in this research is voluntary. If you don’t wish to take part, you don’t have to. You will receive the best possible care whether or not you take part.

If you decide you want to take part in the research project, you will be asked to sign the Participant Consent Form. By signing it you are telling us that you:

• Understand what you have read

• Consent to take part in the research project

• Consent to have the tests and treatments that are described

• Consent to the use of your personal and health information as described.

You will be given a copy of this Participant Information Sheet and Consent Form to keep.

**2 What is the purpose of this research?**

The aim of the project is to investigate whether an investigational probiotic supplement is beneficial in improving blood glucose and lipid profiles in obese participants with pre-diabetes and recently diagnosed with type 2 diabetes. An ‘investigational’ treatment means that this probiotic formulation may or may not provide additional health benefits and may or may not enhance your response to the standard care. We will also be assessing which gastrointestinal bacteria play a role in obesity-related disorders and the therapeutic effect of the probiotic supplement.

Investigations have shown the link between the gastrointestinal tract bacteria and obesity, diabetes, cardiovascular disease and metabolic syndrome. Human studies have revealed that specific probiotic strains may improve glucose tolerance, serum lipids, fat mass, inflammatory markers and systolic blood pressure. Therefore, using these ingredients as a medical supplement may help to improve the clinical manifestations related to pre-diabetes.

The results of this research will confirm whether or not the probiotic supplement at a dose of 2000 mg per day is effective for improving blood glucose levels, inflammatory markers and lipid profile. Stool samples will be required to analyse gastrointestinal bacteria and will be performed and funded by Dr Henry Butt (Bioscreen, Melbourne).

This research has been initiated by the clinical trial investigators, Professor Ian Caterson, Professor Luis Vitetta, Dr Samantha Coulson and the study coordinator, Ms Talia Palacios. The results of this research will be used by Talia Palacios to obtain a PhD degree.

**3 What does participation in this research involve?**

The research will be performed at the Charles Perkins Centre (CPC) – Clinical Research Facility and will assess 60 people with pre-diabetes or those recently diagnosed with type 2 diabetes (within 12 months). In the first instance, you will have your height and weight measured and will be asked to undergo an oral glucose tolerance test (OGTT) which involves drinking a sugary drink and giving three blood samples over a two hour period (at 0, 60 and 120 minutes) to evaluate your fasting glucose and glucose tolerance. If the results of these procedures show that the study is suitable for you, you will be invited to continue in the study.

Eligible participants will be randomly allocated (like the toss of a coin) to take either the probiotic supplement or placebo capsules for 12 weeks. This is a double-blind study, which means that neither you nor your study coordinator will know which treatment you are receiving. The probiotic supplement and the placebo will look, smell and taste the same. However, in certain circumstances your study coordinator can find out which treatment you are receiving, and upon completion of the study, all participants will be told which supplement they were taking.

The supplement has to be taken with cold non-carbonated water. The dose is 2 capsules twice per day (before breakfast and dinner). **The product should not be mixed or taken with hot drinks or foods as the heat can inactivate the probiotic bacteria.** If you forget to take the probiotic supplement, do not try to make up for missed doses by taking an extra dose. This may increase the chance of getting an unwanted side effect. If it is almost time for your next dose, skip the dose you missed and take the next dose when you are meant to. You will need to record the number of capsules taken each day in a diary provided by us.

In addition, you will be asked to:

- **Provide blood samples** (5 mL) on two occasions. These will be used to measure inflammatory markers, lipids and gut permeability.
- **Provide stool samples** on two occasions. You will be asked to collect these at home. The first stool sample has to be collected before starting to consume the study product. You will be provided with a specialised kit and instructed on how to use it. Once you have your stool sample please bring them back to the CPC. These will be used to measure faecal bacteria.
- **Complete 4 brief questionnaires** (via interview) that assess gastrointestinal symptoms, diet, physical activity and quality of life on 3 occasions. These will take about 30 minutes to do.
- **Use a pedometer** provided by us and record the number of steps, kilometres and calories burned in the study diary.

- **Record your food intake** for 3 consecutive days (2 weekdays and 1 weekend).

In conclusion, you will be required to come to the CPC in 4 occasions.

**4 What do I have to do?**

To participate in the study **you need to avoid eating / drinking yoghurt, fermented food and dietary supplements (fish oil, probiotics, multivitamins minerals, herbal preparations)**. **Antibiotics must also be avoided if possible** while you are participating in the study. If antibiotics are taken the faecal bacterial data will not be used for analysis. However, if your doctor recommends you take antibiotics, please follow your doctors’ instructions but let the study coordinator know as soon as possible if you intend to take these medications. All other prescribed medications will be allowed.

You should take the study product every day and return the remaining product to your study coordinator at your 3rd and 4th visit.

**5 Other relevant information about the research project**

The study will recruit 60 participants, 30 per group. The study will only be conducted at the CPC in Sydney and involves researchers from the Boden Institute of Obesity, Nutrition, Exercise & Eating Disorders (University of Sydney). Investigators from Medlab Clinical Ltd in Sydney and Bioscreen (University of Melbourne) are collaborating with us on this study by providing the study product and expertise in microbial faecal analysis.

**6 Do I have to take part in this research project?**

Participation in any research project is voluntary. If you do not wish to take part, you do not have to. If you decide to take part and later change your mind, you are free to withdraw from the project at any stage.

**7 What are the alternatives to participation?**

You do not have to take part in this research project to receive treatment for your condition. Other options are available; these include receiving normal routine care from your GP, endocrinologist and nutritionist who will continue to provide you with exercise, diet and medication advice to manage your blood glucose levels. Your study coordinator will discuss these options with you before you decide whether or not to take part in this research project. You can also discuss the options with your local doctor.

**8 What are the possible benefits of taking part?**

We cannot guarantee or promise that you will receive any benefits from this research; however, possible benefits may include improved fasting glucose, glucose tolerance, lipid profiles, and obesity-associated inflammation and impaired gut permeability. If you have diarrhoea or other gastrointestinal symptoms associated with the use of metformin you will potentially feel a relief in these symptoms.

**9 What are the possible risks and disadvantages of taking part?**

Medical treatments often cause side effects. You may have none, some or all of the effects listed below, and they may be mild, moderate or severe. If you have any of these side effects, or are worried about them, talk with your study coordinator. Your study coordinator will also be monitoring you for side effects.

Probiotics are not associated with any major side effects and extensive safety data are available on their effects; however minor bloating and flatulence has been reported which may appear during the first few days of consuming the product. It is recommended to take the test product with your meals (breakfast and dinner).

There may be side effects that the researchers do not expect or do not know about and that may be serious. Tell your study coordinator immediately about any new or unusual symptoms that you get. Many side effects go away shortly after treatment ends. However, sometimes side effects can be serious, long lasting or permanent. If a severe side effect or reaction occurs, you may need to stop your treatment. Your study doctor will discuss the best way of managing any side effects with you.

Probiotics are considered safe for pregnant and lactating women, however the effects of the probiotic on the unborn child and on the newborn baby are not known. Because of this, it is important that participants are not pregnant or breast-feeding and do not become pregnant during the research project. You must not participate in the research if you are pregnant or trying to become pregnant, or breast-feeding. If you are a woman and child-bearing is a possibility, you will be required to undergo a pregnancy test prior to commencing the research project. Female participants are strongly advised to use effective contraception during the course of the research.

If you do become pregnant whilst participating in the research project, you should advise your study coordinator immediately. Your study coordinator will withdraw you from the research project and advise on further medical attention should this be necessary. You must not continue in the research if you become pregnant.

Blood collection: Possible risks and discomforts may include slight bruising, discomfort or bleeding from the blood sampling procedures. However, the effects are minor and should not persist beyond a few days.

Oral glucose tolerance testing: During the glucose tolerance test you might feel nauseated, sweaty, light-headed, or may even feel short of breath or faint after drinking the glucose. However, serious side effects of this test are very uncommon.

**10 Will participating in the study cost me anything?**

There are no additional costs associated with participating in this research project, nor will you be paid. All medication, tests and medical care required as part of the research project will be provided to you free of charge.

**11 What will happen to my test samples?**

Test samples that will be taken for this study include blood samples and faecal samples which are mandatory for our research purposes. Blood samples will be taken by trained phlebotomists at the CPC and will be used to assess your blood sugar level, lipids, inflammatory markers and gut permeability. We will compare levels of these markers before you begin taking the test products and again after 12 weeks of supplementation with the test products. Blood samples will be handled, analysed and stored according CPC pathology protocol. Blood samples will be identified by a study code. Blood test results will remain confidential and will only be sent to the study coordinator for analysis. Paper copies of the results will be stored in a locked filing cabinet which is only accessible to the research coordinators. Results will be entered into an Excel spread sheet in coded format.

Faecal samples will be collected and packaged by you. Bioscreen microbiologists will receive and handle your sample and prepare it for analysis. The microbiologists will produce a report on the bacterial species they detect in your sample and this report will be emailed only to the study coordinator which will identify you by a code. All results will remain confidential. Paper copies of the results will be stored in a locked filing cabinet which is only accessible to the research coordinators. Bacterial results will be entered into an Excel spread sheet in a coded format. Faecal samples will be disposed of after analyses.

As we require blood samples from you on three occasions, you will also be asked to provide consent for the collection and storage of your blood to use for this research project.

**12 What if new information arises during this research project?**

Sometimes during the course of a research project, new information becomes available about the treatment that is being studied. If this happens, your study doctor will tell you about it and discuss with you whether you want to continue in the research project. If you decide to withdraw, your study doctor will make arrangements for your regular health care to continue. If you decide to continue in the research project, you will be asked to sign an updated consent form.

Also, on receiving new information, your study doctor might consider it to be in your best interests to withdraw you from the research project. If this happens, he/she will explain the reasons and arrange for your regular health care to continue.

**13 Can I have other treatments during this research project?**

Whilst you are participating in this research project, you may not be able to take some or all of the medications or treatments you have been taking for your condition or for other reasons. It is important to tell your study coordinator and the study staff about any treatments or medications you may be taking, including over-the-counter medications, vitamins or herbal remedies, acupuncture or other alternative treatments. You should also tell your study doctor about any changes to these during your participation in the research project. Your study coordinator should also explain to you which treatments or medications need to be stopped for the time you are involved in the research project.

**14 What if I withdraw from this research project?**

If you decide to withdraw from the project, please notify a member of the research team before you withdraw. This notice will allow that person or the research supervisor to discuss any health risks or special requirements linked to withdrawing.

**15 Could this research project be stopped unexpectedly?**

This research project may be stopped unexpectedly for a variety of reasons. These may include reasons such as:

- Unacceptable side effects
- Decisions made in the commercial interests of the sponsor or by local regulatory/health authorities.

**16 What happens when the research project ends?**

After a report of the trial results has been prepared (up to four months or more after you complete the study) you may contact any of the listed contact people to find out the result of the research, including whether the test product was considered beneficial. You may also request your laboratory test results. If the study results demonstrate that the probiotic supplement was significantly effective in improving glucose management and lipid profiles, you can contact the study coordinator to get information about the product. If you do not wish to take the probiotic, you will continue with your normal routine of care.

**17 What will happen to information about me?**

By signing the Participant Consent Form you consent to the study doctor and relevant research staff collecting and using personal information about you for the research project. Any information obtained in connection with this research project that can identify you will remain confidential. All paper documents will be kept in a locked filing cabinet in the study coordinators office. Staff only have access to the building and the office. Data will be completed on source notes (both written on paper and time and date stamped electronic capture) and entered into an eCRF database at The Boden Institute. This database will be password protected and backed up on The University of Sydney server. Your information will only be used for the purpose of this research project and it will only be disclosed with your permission, except as required by law.

Your health records and any information obtained during the research project are subject to inspection (for the purpose of verifying the procedures and the data) by the relevant authorities and authorised representatives of the institution relevant to this Participant Information Sheet and The University of Sydney, or as required by law. By signing the Participant Consent Form, you authorise release of, or access to, this confidential information to the relevant study personnel and regulatory authorities as noted above.

It is anticipated that the results of this research project will be published and/or presented in a variety of forums. In any publication and/or presentation, information will be provided in such a way that you cannot be identified. Data will be represented by participant numbers and groups (treatment or placebo) and individual results will not be published or presented.

In accordance with relevant Australian privacy and other relevant laws, you have the right to request access to your information collected and stored by the research team. You also have the right to request that any information with which you disagree be corrected. Please contact the study team member named at the end of this document if you would like to access your information. Any information obtained for the purpose of this research project that can identify you will be treated as confidential and securely stored. It will be disclosed only with your permission, or as required by law.

**18 Complaints and compensation**

If you suffer any injuries or complications as a result of this research project, you should contact the study team as soon as possible and you will be assisted with arranging appropriate medical treatment. If you are eligible for Medicare, you can receive any medical treatment required to treat the injury or complication, free of charge, as a public patient in any Australian public hospital.

In the event of loss or injury, the parties involved in this research project have agreed to compensation through the University of Sydney.

**19 Who is organising and funding the research?**

This research project is being conducted by Professor Ian Caterson, Professor Luis Vitetta, Dr Samantha Coulson, Dr Henry Butt and Ms Talia Palacios. The probiotic and the funding to pay for laboratory supplies and pathology tests will be donated by Prof Luis Vitetta (Director of Medical Research for Medlab Clinical Ltd. and affiliated with the University of Sydney) to the Boden Institute of Obesity, Nutrition, Exercise & eating Disorders, The University of Sydney.

By taking part in this research project you agree that samples of your blood or tissue (or data generated from analysis of these materials) may be provided to The University of Sydney.

The University of Sydney may directly or indirectly benefit financially from your samples or from knowledge acquired through analysis of your samples.

You will not benefit financially from your involvement in this research project even if, for example, your samples (or knowledge acquired from analysis of your samples) prove to be of commercial value to The University of Sydney.

In addition, if knowledge acquired through this research leads to discoveries that are of commercial value to The University of Sydney, the study researchers or their institutions, there will be no financial benefit to you or your family from these discoveries.

No member of the research team will receive a personal financial benefit from your involvement in this research project (other than their ordinary wages).

**20 Who has reviewed the research project?**

All research in Australia involving humans is reviewed by an independent group of people called a Human Research Ethics Committee (HREC). This study has been approved by the Ethics Review Committee (RPAH Zone) of the Sydney Local Health District. Any person with concerns or complaints about the conduct of this study should contact the Executive Officer on 02 9515 6766 and quote protocol number X14-0369.

This project will be carried out according to the *National Statement on Ethical Conduct in Human Research (2007, updated March 2014)*. This statement has been developed to protect the interests of people who agree to participate in human research studies.

**21 Further information and whom to contact**

If you want any further information concerning this project or if you have any medical problems which may be related to your involvement in the project (for example, any side effects), you can contact the principal study researcher Luis Vitetta on 02 8188 0311 or 04 0226 3316 or:

**Clinical Coordinator contact person**

Name Talia Palacios (PhD scholar)

Position Primary investigator

Telephone 04 5018 0801

Email [tpal5781@uni.sydney.edu.au](mailto:tpal5781@uni.sydney.edu.au)

For 24-hour medical contacts, please contact Talia Palacios on 02 8627 1962 or 04 5018 0801


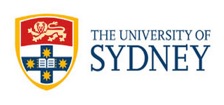


**Participant Consent Form**

| **Title** | The Effect of a Multistrain Probiotic on Metabolic Biomarkers in Adults with Pre-diabetes and Recently Diagnosed with Type 2 Diabetes. |
| --- | --- |
| **Protocol Number** | X14-0369 |
| **Coordinating Principal Investigators** | Prof Ian Caterson  Prof Luis Vitetta |
| **Associate Investigator(s)** | Ms Talia Palacios  Dr Samantha Coulson  Dr Henry Butt |
| **Location** | Sydney |

**Declaration by Participant**

I have read the Participant Information Sheet or someone has read it to me in a language that I understand. I have had an opportunity to ask questions and I am satisfied with the answers I have received.

I understand the purposes, procedures and risks of the research described in the Participant Information Sheet.

I give permission for my doctors, other health professionals, hospitals or laboratories outside this hospital to release information to The University of Sydney concerning my disease and treatment for the purposes of this project. I understand that such information will remain confidential.

I freely agree to participate in this research project as described and understand that I am free to withdraw at any time during the study without affecting my future health care.

I understand that I will be given a signed copy of this document to keep.

|  | | | | | | |
| --- | --- | --- | --- | --- | --- | --- |
|  | Name of Participant (please print) | |  |  |  |  |
|  | | | | | | |
|  | Signature |  | | Date |  |  |
|  | | | | | | |

|  | | | | | | |
| --- | --- | --- | --- | --- | --- | --- |
|  | Name of Witness to Participant’s signature (please print) | |  |  |  |  |
|  | | | | | | |
|  | Signature |  | | Date |  |  |
|  | | | | | | |

**Declaration by Study Coordinator**

I have given a verbal explanation of the research project, its procedures and risks and I believe that the participant has understood that explanation.

|  | | | | | | |
| --- | --- | --- | --- | --- | --- | --- |
|  | Name of Study Coordinator (please print) | |  | | |  |
|  | | | | | |  |
|  | Signature |  | | Date |  |  |
|  | | | | | | |

Note: All parties signing the consent section must date their own signature.

I understand that, if I decide to discontinue the study treatment, I may be asked to attend follow-up visits to allow collection of information regarding my health. Alternatively, a member of the research team may request my permission to obtain access to my medical records for collection of follow-up information for the purposes of research and analysis.

I consent to the use of blood and tissue samples taken from me, as described in the relevant section of the Participant Information Sheet, for this specific research project.

|  | | | | | | |
| --- | --- | --- | --- | --- | --- | --- |
|  | Name of Participant (please print) | |  | | |  |
|  | | | | | | |
|  | Signature |  | | Date |  |  |
|  | | | | | | |

|  | | | | | | |
| --- | --- | --- | --- | --- | --- | --- |
|  | Name of Study Coordinator (please print) | |  | | |  |
|  | | | | | |  |
|  | Signature |  | | Date |  |  |
|  | | | | | | |

Note: All parties signing the consent section must date their own signature.


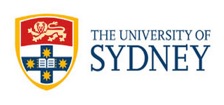


**Form for Withdrawal of Participation**

| **Title** | The Effect of a Multistrain Probiotic on Metabolic Biomarkers in Adults with Pre-diabetes and Recently Diagnosed with Type 2 Diabetes. |
| --- | --- |
| **Protocol Number** |  |
| **Coordinating Principal Investigators** | Prof Ian Caterson  Prof Luis Vitetta |
| **Associate Investigator(s)** | Ms Talia Palacios  Dr Samantha Coulson  Dr Henry Butt |
| **Location** | Sydney |

**Declaration by Participant**

I wish to withdraw from the above research project and understand that such withdrawal will not affect my routine treatment, my relationship with those treating me or my relationship with The University of Sydney.

|  | | | | | | |
| --- | --- | --- | --- | --- | --- | --- |
|  | Name of Participant (please print) | |  |  |  |  |
|  | | | | | | |
|  | Signature |  | | Date |  |  |
|  | | | | | | |

Reasons for withdrawing from the research project (*only record if volunteered by the participant*)

|  |
| --- |

**Declaration by Study Doctor/Senior Researcher^†^**

I have given a verbal explanation of the implications of s from the research project and I believe that the participant has understood that explanation.

|  | | | | | | |
| --- | --- | --- | --- | --- | --- | --- |
|  | Name of Study Coordinator (please print) | |  | | |  |
|  | | | | | |  |
|  | Signature |  | | Date |  |  |
|  | | | | | | |

Note: All parties signing the consent section must date their own signature.
